# Investigation of fast and efficient lossless compression algorithms for macromolecular crystallography experiments

**DOI:** 10.1107/S160057752400359X

**Published:** 2024-06-05

**Authors:** Herbert J. Bernstein, Jean Jakoncic

**Affiliations:** a Ronin Institute for Independent Scholarship, c/o NSLS-II Brookhaven National Laboratory Bldg 745 Upton NY11973-5000 USA; b National Synchrotron Light Source II Brookhaven National Laboratory Bldg 745 Upton NY11973-5000 USA; University of Tokyo, Japan

**Keywords:** macromolecular crystallography diffraction, lossless compression, high framing rate

## Abstract

Structural biology experiments benefit significantly from state-of-the-art synchrotron data collection. One can acquire macromolecular crystallography (MX) diffraction data on large-area photon-counting pixel-array detectors at framing rates exceeding 1000 frames per second, using 200 Gbps network connectivity, or higher when available. In extreme cases this represents a raw data throughput of about 25 GB s^−1^, which is nearly impossible to deliver at reasonable cost without compression. Our field has used lossless compression for decades to make such data collection manageable. Many MX beamlines are now fitted with DECTRIS Eiger detectors, all of which are delivered with optimized compression algorithms by default, and they perform well with current framing rates and typical diffraction data. However, better lossless compression algorithms have been developed and are now available to the research community. Here one of the latest and most promising lossless compression algorithms is investigated on a variety of diffraction data like those routinely acquired at state-of-the-art MX beamlines.

## Abstract 2

Many macromolecular crystallography beamlines are now fitted with DECTRIS Eiger detectors, which are all delivered with optimized compression algorithms by default; they perform well with current framing rates and typical diffraction data. However, better lossless compression algorithms have been developed and are now available to the research community.

## 1. Introduction

**Figure 1:**
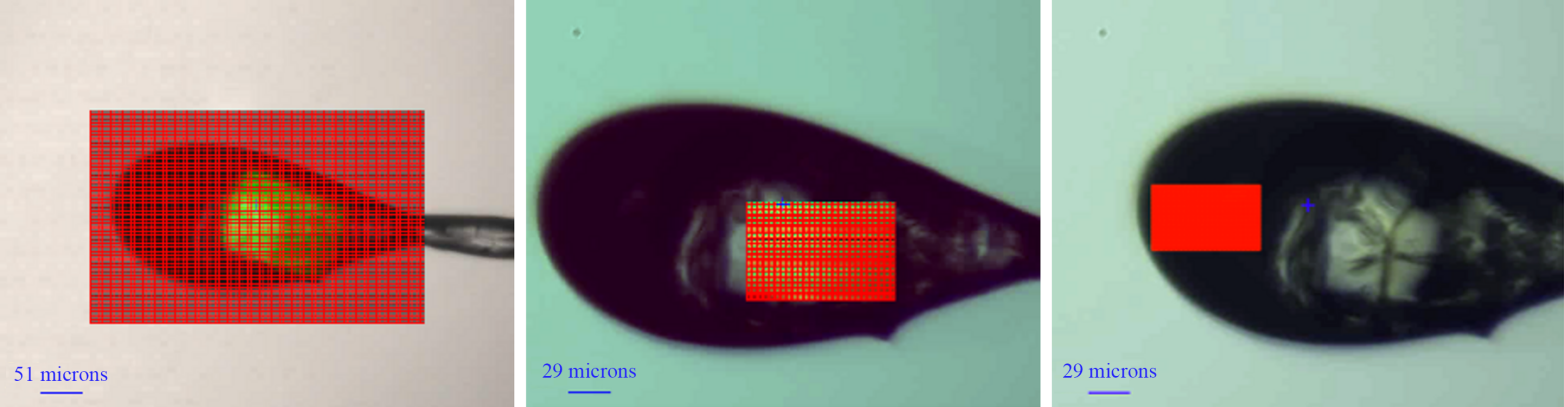
Screenshots of the raster area used for the experiment; images captured from the live video feed of the on-axis microscope section of the Life Sciences Data Collection application, LSDC (Hill *et al.*, 2020 ▸). Left: large area raster covering an area larger than the loop. The same area was used for collections LTL.5 to LTL25, using different beam intensities reflected in the beam transmission. Center: raster area over the crystal only, all frames with diffraction from the single crystal from collection CO5. Right: raster area over the solution only for collection SO5; none of the frames in collection SO5 have diffraction from the crystal. The left-hand image was acquired using the low-magnification branch of the AMX on-axis microscope. The center and right-hand images were acquired using the high-magnification branch.

Modern macromolecular X-ray crystallography is a particularly demanding application of data compression. Data collection involves a detector collecting hundreds to thousands of 4–17 megapixel images per second. Because experimenters can never be sure that any particular specimen will produce useful diffraction data, and because the whole process is quick, they will measure data from many specimens. Modern MX beamlines have implemented preliminary raster scanning, allowing users to scan a sample rapidly to locate the diffracting sample or the best diffracting volume in the sample, before collecting the real data; see Figs. 1 ▸ and 2 ▸. Every image is compressed before it is written to any storage medium.

**Figure 2:**
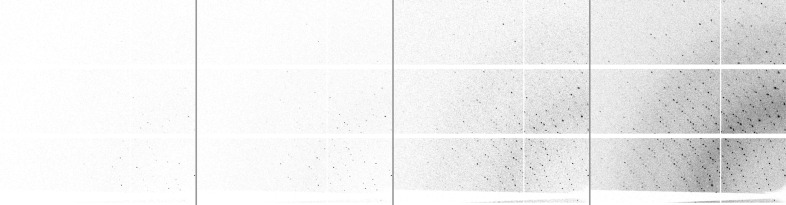
Diffraction pattern of the same area of the larger-than-loop raster with increasing beam intensity. For all diffraction patterns the sample was exposed for 5 ms, with an oscillation of 0.05° at 13.5 keV. From left to right: the sample was exposed to 0.5%, 1%, 5% and 25% of the full beam intensity at AMX, which was 4.35 × 10^12^ photons s^−1^ at the time of these experiments.

Useful images that can provide the information needed to solve macromolecular structures show hundreds to thousands of diffraction spots in a pattern covered mostly with scatter background. Inadequate specimens produce images that, to the eye of a non-expert, are similar and therefore must be examined. The dynamic ranges of the diffraction spots (Bragg, 1912 ▸) can be quite large, but the images are mostly ‘empty’ and usually benefit from compression (Fig. 2 ▸).

Common practice in crystallography has been to focus on so-called ‘lossless’ compression algorithms for diffraction images, *i.e.* on algorithms that preserve the entire information content of the image in the sense discussed by Claude Shannon (Shannon, 1948 ▸) in his ‘source coding theorem’ which tells us that we cannot compress the image to a size smaller than the Shannon entropy limit. The entropy limits presented in this paper were determined experimentally with multiple lossless compression algorithms. The potential impact of lossy compression (*i.e.* compression to a smaller size than the entropy limit at the cost of loss of information) will be discussed in subsequent papers. There are MX experiments for which either science or policy or both argue for preservation of the raw data behind a publication. Available resources are not sufficient to allow one to err on the side of caution and to retain all data in all cases. Current data management policy is changing. More data will have to be retained, eventually requiring more powerful compressions, possibly including lossy compression. In this report we confine our attention to the lossless case, which preserves all the data; at the cost of having to choose which experimental data to keep.

There are many effective approaches to the lossless compression of digital images in general; see Rahman & Hamada (2019 ▸) for a recent review. The algorithms related to the Lempel–Ziv–Welch compression algorithm (LZW) (Ziv & Lempel, 1977 ▸; Welch, 1984 ▸) are, in many cases, the best choice for a wide range of applications. However, LZW and its derivative algorithms were not implemented for MX data because of an active patent (Welch, 1985 ▸), which would have resulted in significant added costs. As a result, for decades the MX field employed other algorithms (Abrahams, 1993 ▸; Hammersley, 1996 ▸; Ellis & Bernstein, 2015 ▸; Bernstein *et al.*, 1999 ▸).

The first commercial hybrid pixel array detector, the Dectris Pilatus 6M (Brönnimann *et al.*, 2003 ▸), adopted the CBF format with byte-offset compression (Bernstein, 2010 ▸). This compression was adequate at the time, given the detector framing rate and its number of pixels. Fast-forward a few years and Dectris developed the next generation of hybrid pixel-array detectors, the Eiger detector. The largest Eiger detector, the 16M, runs more than ten times faster and has three times the number of pixels, generating at least 30 times the data throughput. Further increases in data rates are appearing due to beamline upgrades and use of faster charge-integrating detectors in place of conventional photon-counting pixel detectors (Grimes *et al.*, 2023 ▸). Given the progress in beamlines and detectors, upgraded compression algorithms are urgently needed.

By 2012, the LZW patents had expired, and available computer hardware could handle higher data-framing rates and more complex compressions. In 2013 Dectris designated Collet’s LZ4 compression (Collet, 2011 ▸), a very fast byte-oriented version of Lempel–Ziv compression without an entropy stage, as the compressor for the Eiger (Donath *et al.*, 2013 ▸). Entropy coding is best described as a lossless data compression that represents frequently occurring patterns with shorter codes and sparse patterns with longer codes, thereby reducing the overall size of the compressed data. The entropy coding step follows dictionary-based compression found in many algorithms, including DEFLATE (used in gzip and zlib).

Later the compression ratios were improved by adding a pre-conditioning stage using Masui’s bit-shuffle algorithm (Masui *et al.*, 2015 ▸). As discussed in that paper, bit shuffle is descended from the earlier byte-oriented shuffle algorithm. The two-stage compressor is called ‘bslz4’. It has proven to be fast and reliable, but in some cases it has produced data sets that are still significantly more compressible. The alternative two-stage compressor preconditioning with ‘shuffle’ (*i.e.* ‘byte-shuffle’) followed by lz4 is called ‘slz4’. There are some rare cases in which slz4 compresses better than bslz4, but it is not a general solution to the issues with bslz4.

All Dectris Eiger detectors can be operated in two modes, the file-writer mode in which bslz4-compressed HDF5 data files containing anywhere from 50 to 500 individual diffraction patterns are written onto the file system, and the streaming mode where diffraction frames are made available as a zeroMQ (Hintjens, 2013 ▸) stream with or without compression as soon as they are available. For the streaming interface, downstream dedicated applications can digest and process the data, or digest, process and store the data in the form of either CBF or HDF5 files. The Eiger2 detectors now in use on some MX beamlines run four times faster, further supporting the need to revisit compression algorithms to enable improvement.

In the interim, others have developed and experimented with further LZW compression algorithms. A popular, fast and efficient algorithm is Zstandard (‘zstd’) (Collet & Kucherawy, 2021 ▸). The clearest description of Zstandard for a general audience is from Wikipedia (Wikipedia, 2023 ▸), which says, in part, ‘Zstandard was designed to give a compression ratio comparable to that of the DEFLATE (Oswal *et al.*, 2016 ▸) algorithm (developed in 1991 and used in the original ZIP and gzip programs), but faster, especially for decompression. It is tunable with compression levels ranging from negative 7 (fastest) to positive 22 (slowest in compression speed, but best compression ratio). Unlike LZ4, Zstandard includes an entropy compression stage. Compression speed can vary by a factor of 20 or more between the fastest and slowest levels, while decompression is uniformly fast, varying by less than 20% between the fastest and slowest levels (see the supporting information for a compression level description). Zstandard reaches the current Pareto frontier, as it decompresses faster than any other currently available algorithm with similar or better compression ratio.’ Note that when preconditioning with bit-shuffle is used with Zstandard, the various Zstandard compression levels can also be used.

In this paper we report on experiments comparing the speed and effectiveness of lossless compressions: lz4, bslz4, zstd as well as bitshuffle followed by zstd (‘bszstd’) and byte-shuffle followed by zstd (‘szstd’). We also explore the impact of multi-threading on performance. We restrict our consideration to ‘lossless’ compressions that faithfully preserve all the data in the diffraction images. In a future investigation we will also consider the option of ‘lossy’ compressions that allow loss of some of the background information not needed to preserve the accuracy of Bragg reflections.

## 2. Materials and methods

To generate data suitable for compression tests, we used a single large crystal (approximately 150 µm × 100 µm × 75 µm) of hen egg-white lysozyme (HEWL). To obtain data sets of a few thousand images, we employed the rapid raster-scanning mechanism (Fig. 1 ▸), which is used routinely for diffraction-based crystal characterization and centering. We performed this work at the National Synchrotron Light Source II (NSLS-II) Highly Automated Macromolecular Crystallography (AMX) beamline 17-ID-1 (Schneider *et al.*, 2022 ▸) at Brookhaven National Laboratory. Diffraction data were acquired at 13.5 keV, using an Eiger X 9M detector (Förster *et al.*, 2016 ▸). All data were collected in file-writer mode, with each HDF5 data file containing the frames from each raster row. For all data sets in Table 1 ▸ (LTL1, LTL.5, LTL5, LTL25, CO5 and SO5), data were collected with 5 ms exposure time, 5 µm steps for rasters LTL1 to CO5, and 2 µm steps for raster SO5. The data sets are summarized in Table 1 ▸. Comparing the images of Fig. 1 ▸ with Table 1 ▸, one can see that this approach allowed us to collect data with different content – ‘crystal-only’, solvent only and a mixture of good and ‘useless’ images including data from air. Increasing the beam transmission allowed us to record more and stronger reflections. The native focused beam size at AMX is 7 µm × 5 µm, the beam flux is 4.35 × 10^12^ photons s^−1^ and the sample was rotated by 0.05° for each diffraction image. We did not observe any radiation damage that impacted the outcome during the experiment.

Data for this processing experiment were collected using the AMX Dectris Eiger X 9M detector and written on the NSLS-II Luster file system with the default compression bit-shuffle LZ4 (bslz4). A subset of the diffraction patterns, highlighting the intensities of the background and the Bragg reflections is displayed in Fig. 2 ▸. To minimize interference from other operations, we employed a separate computer and ramdisk storage for our tests. We decompressed and then recompressed the data back to ramdisk, with each trial compression using an instrumented h5py (Collette, 2013 ▸) Python script. In all cases the jobs were more than 94% cpu-bound.

We used two parameters to judge the usefulness of each compression method: speed and degree of compression. After we decompressed the original data, we divided the single-thread processor time during recompression by the wall-clock time. The inverse of that divided by the number of images gives a compression speed for each data set in frames per second. This speed should predict the improvement or degradation in frame rates for compression one would achieve if FileWriter were changed to use the trial compression algorithm instead of the default bslz4. We also performed multi-processing using 1, 2, 4, 8, 16, 32, 64 and 96 threads in coarse-grained multiprocessing on a 36 core, 72 thread dual Intel Xeon Gold 6154 CPU with a frequency of 3.00 GHz. The various HDF5 compression filters were accessed via the hdf5plugin package (Vincent, 2021 ▸), using blosc (Alted, 2019 ▸).

## 3. Results

### 3.1. Macromolecular crystallography

**Figure 3:**
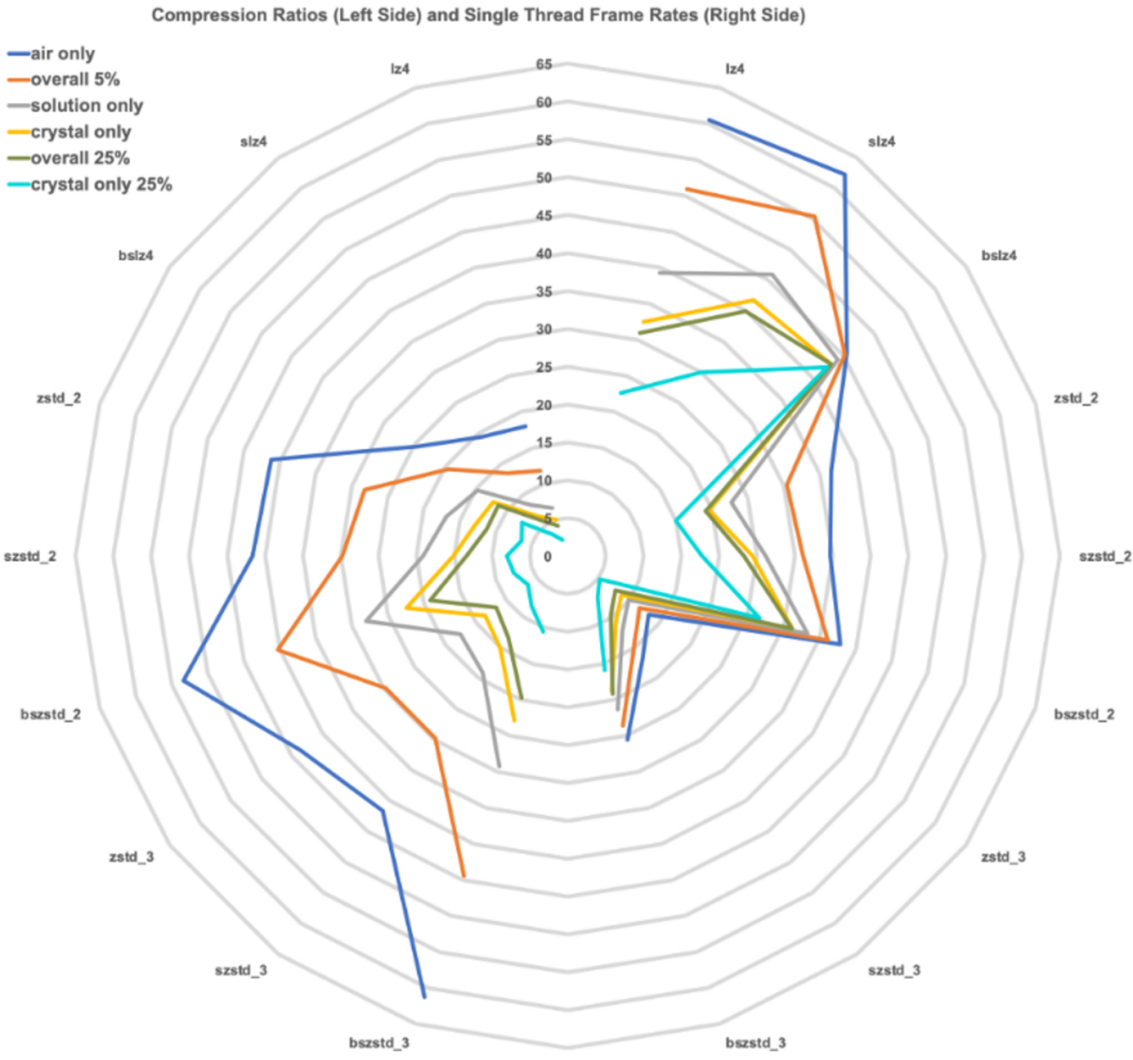
For analysis of the single thread results, the data were organized as ‘air only’ (the first five rows of data set LTL5), ‘overall 25% transmission’ (all of data set LTL25), ‘overall 5% transmission’ (all of data set LTL5), ‘solution only’ (all of data set SO5) and ‘crystal only’ (all of data set CO5). The left-hand side of the figure shows the compression ratio. The right-hand side of the figure shows the single-thread compression frame rates. Note that the root values are the same on both sides, top to bottom, so the left-hand side of the figure relates to the values straight across on the right-hand side.

For analysis of the single thread results, the data were organized as ‘air only’, ‘overall 25% transmission’, ‘overall 5% transmission’, ‘solution only’ (more precisely solution and air) and ‘crystal only’ (more precisely crystal, solution and air). Those results are shown in Table 2 ▸ and Fig. 3 ▸ for frame rates and the achieved compression ratios for lz4, bslz4, slz4, zstd_2 (Zstandard at compression level 2), bszstd_2 (bitshuffle-Zstandard at compression level 2), zstd_3 (Zstandard at compression level 3) and bszstd_3 (bitshuffle-Zstandard at compression level 3), and szstd_3 (byte-oriented shuffle-Zstandard at compression level 3). Higher compression levels need increased memory and time to allow for a more thorough search for repeated bit patterns. Compression levels 4, 5 and 6 are also included in Table 2 ▸ for completeness, but show little gain in compression ratios for the large cost in compression speeds. Note that, for all the compression methods, ‘air only’ is more compressible than ‘overall 25% transmission’, which is more compressible than ‘overall 5% transmission’, which is more compressible than ‘solution only’, which is more compressible than ‘crystal only’, which is the most difficult to compress, on average.

Data from the area of the 25% transmission that corresponds to ‘crystal only’ was extracted and was the slowest and the least compressible data in that study. This is an important finding since more and more data sets are collected on smaller samples with higher beam intensity. It is particularly interesting for experiments requiring multiple partial data collection from many crystals, where samples are exposed to a greater dose-per-degree (Bernstein *et al.*, 2020 ▸; Nguyen *et al.*, 2022 ▸; Matsuura *et al.*, 2023 ▸).

If one does not shuffle the bits, the achievable frame rates decline in the same order for each compression method, with lz4 being fastest and bszstd_3 being slowest, but use of bitshuffle greatly reduced the differences in timing, even though it helps only slightly in reducing the difference in compression ratios. Overall, it appears that use of zstd_2, bszstd_2, zstd_3 and bszstd_3 provides noticeable improvements in compressed file sizes over bslz4, in that order. In terms of speed, bszstd_2 achieved over half the data rate of bslz4.

**Figure 4:**
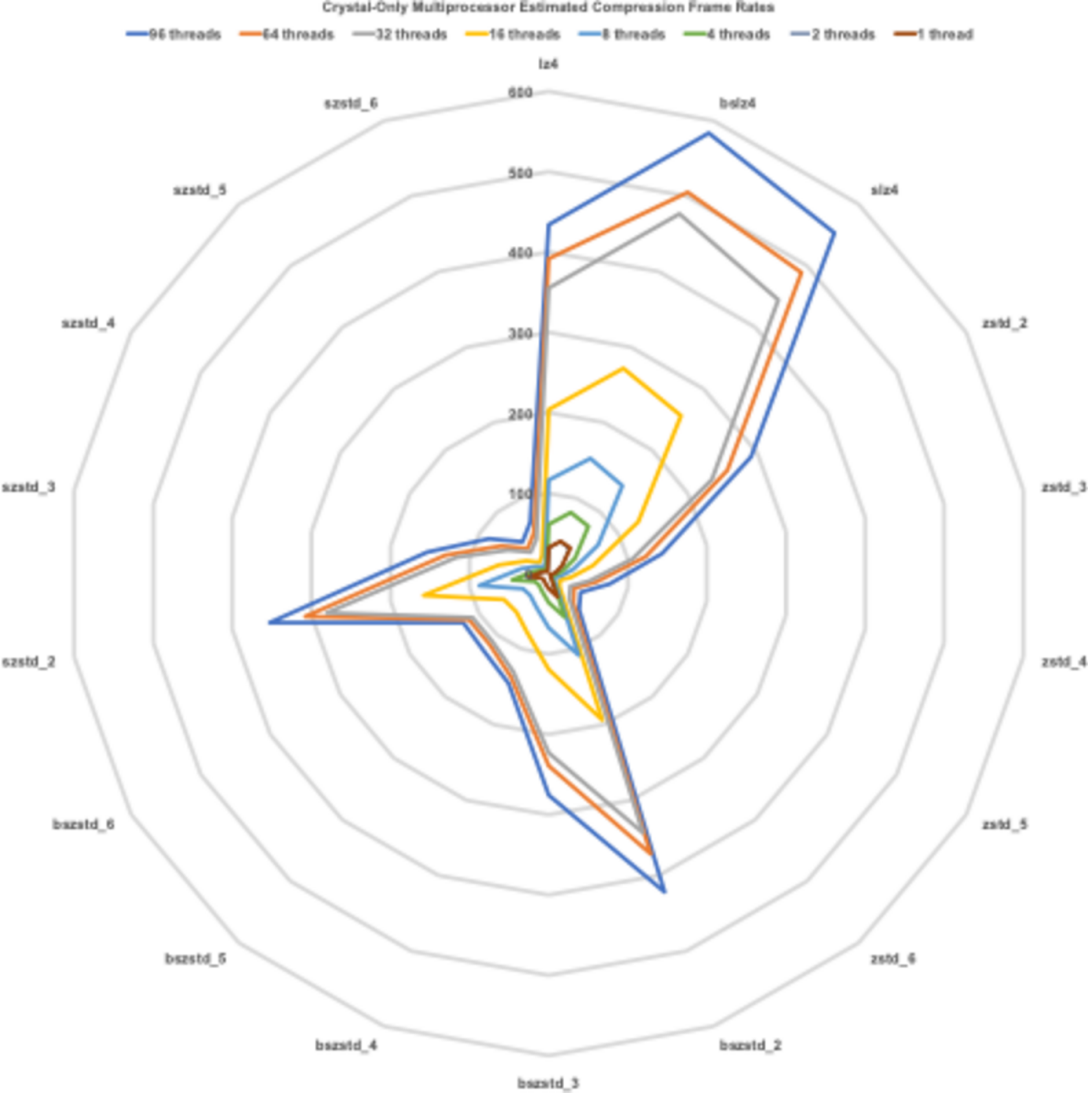
Multiprocessor frame rates achieved for the ‘crystal only’ data (all of data set CO5), using 1, 2, 4, 8, 16, 32, 64 and 96 threads for lz4 and zstd without and with bitshuffle and byte-oriented shuffle. The compression levels for zstd shown are 2, 3, 4, 5 and 6.

We also tested multi-thread compression on our multi-processor computer. Inasmuch as the ‘crystal only’ data are the most difficult to compress, the multi-processor results for data set CO5 indicate most strongly the performance achievable by performing compressions in parallel with one another; see Fig. 4 ▸. For the computer system used for these studies, having 36 cores, the highest frame rates were seen with 96 threads, achieving 583 frames s^−1^ for compression with bslz4, a speedup of 13.6 over the one-thread time, and 421 frames s^−1^ with bszstd_2, a speedup of 13.5 over the one-thread time, and 352 frames s^−1^ with szstd_2, a speedup of 14.5 over the one-thread time. In other words, the speedup is about the same for all methods, *i.e.* linear in the number of threads.

Considering the two extreme cases in this study, *i.e.* air-only with 0.5% of the AMX full flux, and crystal-only with 25% of the AMX full flux, and including the solution-only case with 5% of the beam, as shown in Fig. 3 ▸, air-only is significantly more compressible than either of the other two. The air-only images improved steadily and significantly in compression ratio in the transition from lz4 to slz4 to bslz4 to zstd_2 to bszstd_2. Then there is a slight dip for zstd_3 and szstd_3 with a peak at bszstd_3. The crystal-only 25% transmission images showed no significant improvement in compression ratio after bszstd_2, where it is about one-third as compressible as the air-only images. The 5% transmission solution-only images are less than half as compressible as the air-only images. The behavior of the compression ratios of the hardest-to-compress images in the 25% transmission crystal-only images in data set LTL25 are shown in Table 2 ▸. In every case the bitshuffle version compresses better than just lz4 or just zstd or szstd, and the gain in compression of going beyond bszstd_3 is small but significant. Zstandard higher compression levels use more memory and time to allow it to examine and optimize larger blocks of data. The highest compression levels use the highest memory to cache data to be scanned and compressed to a higher potential level.

Unlike the repetition observed in macromolecular diffraction patterns with Bragg spots, the images from protein solution scattering contain global patterns made of donut-like lobes and distinct rings, possibly impacting achievable compression. Therefore, we considered studying compression on SAXS data.

### 3.2. Small-angle X-ray scattering

**Figure 5:**
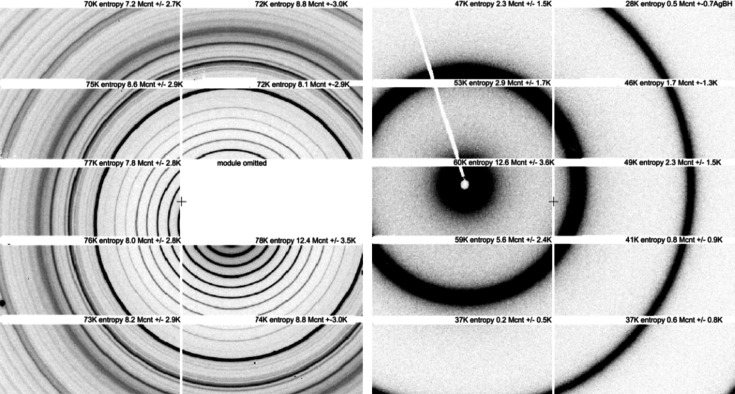
Raw data for SAXS/WAXS for AgBH collected on two Dectris Pilatus 1M detectors with 487 × 195 172 µm pixel modules, nine modules in the left WAXS image, 10 in the right SAXS image (which fits in the space for the missing module in the WAXS image). Entropy limit, total counts and total counts standard deviations are included on each module.

Our NSLS-II beamline that collects SAXS data, the Life Science X-ray Scattering (LiX) beamline (Yang *et al.*, 2020 ▸), employs two Pilatus detectors, with data stitched and packaged in HDF5 format. For example, Fig. 5 ▸ shows raw SAXS/WAXS data for the AgBH^1^ standard sample. The left-hand image is the wide-angle X-ray scattering (WAXS) image (notice the missing module) and the right-hand image is the small-angle X-ray scattering (SAXS) image. This detector lies approximately eight times as far from the specimen as the WAXS data, filling the space left by the missing module. For each detector image, one numbers the modules first vertically from 0 to 4, then horizontally from 0 to 1. Here the top left module is 0, 0 and the bottom right module is 4, 1.

By default, LIX data are compressed using bslz4. Typical compression ratios range from 4:1 (strong scattering) to 13:1 (buffer only scattering). We have applied the same test compressions on the SAXS/WAXS data and included the corresponding table in Table S1 of the supporting information. Like the macromolecular diffraction compression ratios, the reasonable tradeoff (compression ratio and speed) is achieved with bzstd_2. Higher compression levels do not improve the ratio by much and consume more resources. As for MX data, the bslz4 default compression at LIX is faster than bszstd_2 but provides less compression and does not achieve the entropy limit for SAXS data. Simply applying zstd_2 to two already bslz4-compressed hdf5 data files of 5.35 megabytes and 30.9 megabytes produced files of 4.8 megabytes and 27.8 megabytes, respectively, an 11% improvement. One achieves slight additional improvements for the SAXS data by use of high zstd compression levels.

Even though the rings in each module image look simple and uniform, the entropy limits, module-by-module for the WAXS image, range from 70 to 78 kB (2.7 to 2.5 compression ratio, respectively); the details of information in the rings is finer grained than what we can see with our eyes. We need to rely on the compression algorithm to gauge the truth of the matter.

We empirically determined the entropy limit by using the best lossless compression algorithms (bzip2 and zstandard) and logging the minimum achievable file size after compression. For the SAXS image the entropy limits module-by-module are 37 to 60 kB (5.3 to 3.2 compression ratio, respectively), confirming the implication of Shannon’s source coding theorem that the limit on lossless compression is the amount of information detail in each area being compressed. Note in particular that the two modules at the bottom of the SAXS image, modules 4,0 and 4,1, having the fewest detailed features of the image and only 0.2 and 0.6 Mcounts, are the most compressible as revealed by the low entropy. Module 3,1 with 12.4 Mounts just below the missing module in the WAXS image has the largest number of information details and is least compressible, despite the major features appearing to be large, superficially smooth ring segments. The fine texture details and large number of counts in the rings and background limit the compression.

## 4. Summary and conclusion

Since 2013, the default compression algorithm for Eiger diffraction images has been LZ4. This was improved in 2015 by use of a bitshuffle preprocessing pass, creating bslz4 compression. Since then more efficient compression algorithms, such as Zstandard, and higher performance computers have become available,

In this paper we have compared the performance of bslz4 and bszstd on a range of lyzosyme-crystal diffraction images, using beam intensities ranging from 0.5% to 25% transmission for air, crystal-only and solvent-only. The results of these experiments suggest that one might consider the combination of bitshuffle and zstandard (bszstd) as an alternative to bslz4. For highly compressible data, using bszstd_2 instead of bslz4 generates a relative savings of about 40% in space at a cost of an extra 35% in time, whereas, for the least compressible data, using bszstd_2 instead of bslz4 generates a relative savings of 8% in space at a cost of an extra 140% in time.

One might then conclude that, at the moment, bszstd_2 might be the best overall choice to obtain the fastest reasonably useful compression.

For the archiving of data, the time for compression is less critical than it is in data collection. Thus, for highly compressible data, using bszstd_3 instead of bslz4 generates a relative savings of about 48% in space at a cost of an extra 176% in time, while for the least compressible data using bszstd_3 instead of bslz4 generates a relative savings of 9% in space at a cost of an extra 540% in time.

For archiving purposes, emphasis should be put on the compression ratio instead of the best compromise between compression ratio and speed. The best compromise is bszstd_3, where compression is nearly plateauing while performance remains useful. Note that zstd5 and bszstd_5 are noticeably slower than zstd6 and bszstd_6.

The impact of lossy compression and custom-made algorithms will be discussed in subsequent papers.

## 5. Related literature

The following references, not cited in the main body of the paper, have been cited in the supporting information: Bernstein (2016 ▸); Bernstein & Goldstein (2023 ▸); Hartley (1928 ▸); Nyquist (2024 ▸).

## Supplementary Material

Sections S1 to S9. DOI: 10.1107/S160057752400359X/ay5624sup1.pdf

## Figures and Tables

**Table 1:** Lysozyme data sets. A 5 ms exposure time was used for each frame, in a raster-scan protocol that alternates the scan direction on successive rows ‘Transmission’ means the fraction of the total X-ray beam produced by the beamline that we allow to illuminate the specimen. The CO25 set is an extract from the LTL25 set where the larger average per-frame size for CO25 highlights the difficulty in compressing the corresponding crystal-only portion of the raster scan. The size range for the CO25 set does not overlap the size range for the whole LTL25 set because the average size for the whole set are row averages which, including large non-crystal areas, are much smaller than the crystal-only average sizes. Each data file contains all diffraction frames from individual rows. The term ‘MB’ refers to 1048576 bytes.

Data set	Area	Transmission	Frames/row × rows	hdf5 data file size min–max MB	Average size range per frame MB
LTL.5	Larger than loop	0.5%	83 × 53	35–52	0.42–0.62
LTL1	Larger than loop	1%	83 × 53	43–71	0.5–0.85
LTL5	Larger than loop	5%	83 × 53	67–115	0.81–1.39
LTL25	Larger than loop	25%	83 × 53	117–189	1.41–2.27
CO5	Crystal only	5%	61 × 41	90–100	1.48–1.64
CO25	Crystal only	25%	18 × 16	NA	2.44–2.78
SO5	Solution only	5%	45 × 27	59–61	1.31–1.36

**Table 2:** Compression ratio (defined as uncompressed data size divided by final compressed file size, in the same units) and compression speed (expressed in frames per second) for three representative data sets The worst compression ratios for the crystal-only portion of 25% transmission larger-than-loop data set LTL25 (CO25) are also the worst compression ratios of the entire study; see the first column. These are the images with the most information. Two other representative sets are shown: LTL5 which corresponds to the more typical compression ratios from the entire rastering data (50% of frames with air only, 30% of frames with solution only and 20% of frames with crystal only) with 5% transmission and the solution only data set SO5 that was also collected with 5% transmission. The compression frame rates for these three data sets and all compressions tested are also given.

	Compression ratio			Compressed frames per second		
Compression	CO25	LTL5	SO5	CO25	LTL5	SO5
lz4	2.21	11.86	6.66	22.59	50.91	39.26
bslz4	7.50	19.50	14.70	42.35	45.21	44.12
slz4	3.53	13.48	8.26	29.91	55.42	45.91
zstd_2	6.42	28.19	16.86	15.00	30.31	22.72
bszstd_2	7.50	40.25	29.25	42.35	36.08	33.29
szstd_2	8.04	29.78	19.08	17.68	31.10	26.14
zstd_3	6.49	29.69	17.56	5.22	11.70	9.87
bszstd_3	10.55	44.43	29.25	15.84	23.57	21.34
szstd_3	8.17	33.48	20.59	8.76	14.85	12.22
zstd_4	7.14	32.34	19.54	2.13	7.18	5.24
bszstd_4	10.78	49.42	31.48	10.90	15.88	14.25
szstd_4	8.77	37.68	23.45	2.86	7.92	5.90
zstd_5	7.31	33.62	20.33	1.07	4.84	3.17
bszstd_5	10.83	50.55	31.95	9.00	12.35	10.93
szstd_5	8.96	39.55	24.50	1.43	5.25	3.49
zstd_6	7.37	33.50	20.13	1.62	6.02	4.22
bszstd_6	10.90	52.03	32.71	9.64	14.15	12.82
szstd_6	9.01	39.36	24.27	2.23	6.58	4.68
Mean	8	34	22	13	21	18

## Footnotes

1 Silver behenate, or AgBH, is the silver salt of a long-chain fatty acid.
