# Supplementary material for: Investigation of fast and efficient lossless compression algorithms for macromolecular crystallography experiments
Source: J Synchrotron Radiat. 2024 Jun 5;31(Pt 4):647–54. doi: 10.1107/S160057752400359X (PMC11226158; doi:10.1107/S160057752400359X)
Supplement: Supplementary file 1 [file s-31-00647-sup1.pdf]

JOURNAL OF SYNCHROTRON RADIATION

Volume 31 (2024)

Supporting information for article:

Investigation of fast and efficient lossless compression algorithms for macromolecular crystallography experiments

Herbert J. Bernstein and Jean Jakoncic

The data for this experiment are deposited in Zenodo (10.5281/zenodo.7887840). The scripts have been deposited on GitHub in http://github.com/nsls-ii-mx/raster_timing.git. At present the scripts are specialized to the particular test cases presented here. However, we welcome contributions of variant scripts to support detectors other than the Eiger X 9M, and other potential compressions, as well as other computing environments. Zstandard is available for a wide range of computer hardware, but timing will, of course, depend on the exact choice of hardware and the use of compilers optimized for that hardware, as well as on the compressibility of the data. Thus one might best install compression plugins built-from-source for the target computers, rather than generic binaries. In this case the timing reported is for code compiled with "gcc (GCC) 8.5.0 20210514 (Red Hat 8.5.0-15)". The instructions on how to recompile the source code of hdf5plugin are available at http://www.silx.org/doc/hdf5plugin/latest/install.html.

We, along with the crystallographic community, are eager to see contributions not so much from experts in the MX field but rather from data-compression enthusiasts and specialists to see whether or not compression ratios and compression speed (expressed in frames per second per GHz-core) can be optimized further using specialized and homebrew codes. We encourage students to explore compression algorithms and efficiency with this data set.

S1. The Basics of Compression

Crystallographic diffraction images are managed in computers in the same manner as blocks of text. *i.e.* as sequential streams of zeros and ones (bits), subject to the rules of "information theory" as developed by Nyquist (Nyquist, 1924), Hartley (Hartley, 1928), and Shannon (Shannon, 1948), especially Shannon's source coding theorem. Whether we are representing images, text, or any other information, the size the stream of bits necessary to faithfully reproduce the original information cannot be smaller than the "entropy" of that information.

There are many available presentations of the concept of entropy. The following is taken from some data communications lecture notes used for teaching about data communications in the 1980s (Bernstein & Goldstein, 2023).

S2. Data Communications Line Handling

In this section we will consider the fundamental building block of networks: the data communications line, which is a device able to accept information at one point and deliver that same information at some distant point. A line may be realized in many ways. One might write letters and send them through the post. One might send pulses over a wire. One might use radio waves, light, sound, or any other medium which is subject to controlled changes in state. A medium may allow

more than one point of delivery for the same information, as with radio broadcasts, or allow only a single point of delivery, as with carrier pigeons. There are advantages and disadvantages in each approach.

Unfortunately, all media are subject to errors and have some limit on their capacity to carry information. One must arrange to detect errors by adding redundant information to the traffic, and one must devise protocols which correct such errors. Data must not be presented faster than it can be handled. Economy dictates that provisions be made for handling more than one data stream on a given line.

To address such problems, we will draw on the tools of information theory, coding theory, elementary physics, and the study of cooperating parallel processes. We will see that we can carry arbitrarily detailed information at any desired small, but non-zero, probability of transmission error, assuming we can provide sufficient information capacity in the line. We will examine codes of varying degrees of efficiency at detecting errors, with particular emphasis on cyclic codes, and we will consider protocols which respond to the errors detected without duplicating or dropping messages. When we are done, we will be able to assume communications lines which are sufficiently reliable to allow us to piece together communications networks.

S3. Data Representation

Quantum physics aside, we live in an effectively continuous world. Interactions with computers require mapping continua of data into discrete and usually finite sets. For example, the human voice is capable of many subtle and expressive sounds. In dealing with computers, we must perforce lump all the ways one might say, for example, “I love you,” into ten (yes, ten) characters. When the intended recipient gets those words, he or she could map them back into any of a wide range of sentiments. We might try to improve the faithfulness of this data representation by using more characters per sentiment. We might say, “I love you very much,” or “I love you like a sister,” or “How do I love thee/ Let me count the ways/ ... “. But, no matter how many words we use, it is doubtful that we will achieve an accurate representation of the original thought.

Since we cannot solve the problem, let us accept it and describe it. The basic model we will use in dealing the problem at hand consists of five elements: Information Source, Outgoing Encoding Device [encoder], Signal Transmission Channel, Incoming Decoding Device [decoder], and Information Destination.

The encoder translates the information from the source to a form suitable for transmission over the channel. For example, the speech centers of our brains translate complex feelings into sequences of sounds to be transmitted by the air. The decoder inverts this translation for the destination. We say that the source information is represented on the channel by the encoded source

information and at the destination by the decoded channel information. [Here] we are concerned with the considerations in representing information.

S4. Defining Information

What do we mean by information? A rose is a rose, not the letters r-o-s-e. For concrete objects, the object itself is the true information. We associate words, i.e. labels, with various objects, so that others can distinguish the objects we have in mind from other objects. The presence of a rose is a particular state of some part of the universe. By information we mean a set of labels associated with various states of some system. If we apply one label to a wide range of states, we give up the ability to distinguish among those states. Conversely, if we wish to preserve the detailed information about many states, we need many distinct labels. If the states change with time, so must the labels. If we cannot change our labels in synchrony with the changes in states, more information will be lost.

In the real world, states can change at arbitrary times, arbitrarily often. In computers and communications systems, we usually limit state changes to certain discrete times (”clock ticks”) or place a lower bound on the time between states changes, or do both. In either case, in any finite time interval we can expect only a finite number of distinct values to be assumed by any labelling states, r(t), in R. In modern digital circuitry, only a finite number of possible values may be assumed by all r in R for all time. Thus, in the clocked case of synchronous digital circuitry, in any finite time interval we have a finite R. In the unclocked case, and in analogue circuitry we might well have an infinite, even uncountable R, but would have no way to distinguish among more than a finite number of classes of states if any synchronous digital circuitry were interposed between the observer and the unclocked or analogue circuitry. Thus it is reasonable to restrict our attention to finite R. (The infinite case does occur in some systems, causing great difficulty in analysing and removing problems).

Given a finite R, the mapping from S to R defines a finite number of equivalence classes on S, by considering two states in S to be in the same class if they are mapped to the same member of R. Two such states, s1 and s2 mapped to the same r, will be indistinguishable after transmission. That we do or do not consider a particular representation sufficiently faithful comes down to our willingness to accept the idea that s1 may be confused with s2.

S5. Binary Representation

Once we accept a particular representation of our data, we can translate that representation into another representation going from S into streams of binary digits. One simple way would be to number the r(t) in R from 1 through card(R), the cardinality of R, i.e. the number of things in R. However, it is more efficient to use 0 through card(R)-1, instead, and take the binary representation of the ordinal of any r(t) in place of that r(t), using leading zeroes, say, to avoid confusion about where one number ends and the next begins. We will need the logarithm to the base 2 of card(R) bits in the

full time interval under consideration. Since the only base we will use for logarithms will be 2, we will use log(card(R)) as our notation. This, of course, leaves us with fractional bits in most cases. For most purposes, we must take a ceiling function to get the next whole number of bits not less than the log. However, when combining statistical aggregates of counts of bits to estimate a required capacity, it may be appropriate to retain the fractions as such.

Suppose we now divide the time interval on a clock tick. At worst then R is the product set of the range of states R1 for interval T1 and the range of states R2 for interval T2. We will need no more than log(card(R1))+log(card(R2)) bits to represent the data, and this value is greater than or equal to log(card(R)). With states uniformly populated in time, then, log(card(R))/T bits per unit time is a reasonable measure of the rate at which information is to be represented.

For example, suppose the range, R, has two possible states in the interval T1 and two possible states in the interval T2:

|<--------T1-------->|<--------T2-------->|

state1	state1	r1
state1	state2	r2
state2	state1	r3
state2	state2	r4

For each of the two states assumed in interval T1, we may assume at most two states in interval T2, for a maximum possible range of states of four, the product of two and two. Since the log of a product is the sum of the logs of the quantities being multiplied, and each interval requires only one bit, the total range requires at most two. If we add another interval allowing two states, we would again multiply the number of possible states by two and add one to the number of bits required. As long as there is no reason to assume that some time interval favors one state or the other, the number of bits required will go up linearly in time.

S6. Escape Codes

In some cases, however, we can reduce this rate because we know that some states are more likely than others, e.g. e’s are more likely than q’s in English text. We could use short bit streams for the most likely states and reserve a short bit stream to indicate that a long bit stream follows for the

less likely cases. This technique of escape sequences is used both to reduce information rate demands, and to expand existing representations to carry more information.

For example, suppose we have an English language text which must also carry some words in a foreign alphabet. We could do a reasonable job for English in a little over 100 characters, requiring seven bits per character. The other alphabet might require, say, 63 additional characters. Thus the total alphabet for both languages would need 163 characters, or eight bits. If the use of those characters is limited to, say, one percent of the text, we could add one special escape character, #, to our English alphabet to indicate that the next character was not an English character, but a transliteration from the foreign alphabet into English. The total text would grow in length by one percent for all the #'s, but, because we could use seven bits per character rather than eight, would be 7.07/8 the length of the more obvious representation.

As a general approach, order the states by decreasing probability, p[i], i = 1,..,card(R). Suppose p[1],..,p[k1] are all greater than or equal to $(1/2)^{l1}$, then since probabilities sum to 1,

$$1 >= p[1]+..+p[k1] >= k1/2^{l1}$$

and we can represent the first k1 states by numbers of no more than l1 bits. Now suppose p[k1+1],..,p[k2] are all greater than or equal to 1/2**l2 which is less than 1/2**l1, then, since

$$1 >= p[1]+..+p[k1]+p[k1+1]+..+p[k2]$$

$$>= k1/2^{l1} + (k2-k1)/2^{l2}$$

we have

$$k2-k1 <= 2^{l2} * (1-k1/2^{l1}) = 2^{(l2-l1)} (2^{l1}-k1)$$

so that we can represent the next k2-k1 states by 2^{l1}-k1 sets of numbers of l2-l1 bits, i.e. we can take each of the unused numbers of l1 bits from our representation of the first k1 states and take that unused number as an escape code flagging a group of l2-l1 bits to follow. Clearly (exercise left to the reader) we can continue this process, so that the unused numbers in this set become escape codes for the next lower probabilities, giving us an expected number of bits in the time interval of

$$(p[1]+..+p[k1])*l1+(p[k1+1]+..+p[k2])*(l1+l2-l1)+..$$

from which the general expression for the number of bits required H,

$$H = \sum_{i=1}^{card(R)} p[i] * \log(1/p[i])$$

The quantity H is called "entropy" because it is of the same form as the expression for entropy used in statistical mechanics, where it is a measure of the disorder of a system. In terms of

information, the more disordered a system, the more distinct messages it can convey, i.e., the more information, and the more bits required.

S7. Entropy

Let us examine the expression for entropy. Notice that all the terms of the sum are nonnegative, as one might expect, and that the only way to achieve an entropy of zero would be to have some state with probability one and all other states with probability zero. In that case, there is only one state, and no bits need be sent to distinguish among states.

In practice, while this extreme information theoretic limit would save bits, it is not worth the coding complexity, and most representations are chosen by other criteria.

Here, we empirically determined the entropy limit by using the best lossless compression algorithms and logging the minimum achievable file size after lossless compression was applied. BZIP2 and Zstandard with high compression levels were used.

S8. Lossless or Faithful Compression

In simplified terms, lossless or faithful compression is thus a matter of examining the bit stream for bit patterns and representing the most frequently occurring patterns by the most compact representations. The more carefully we examine the bit stream, the better our chances of getting close to the entropy limit, so achieving better compression ratios is usually a matter of expending more cpu time and memory. For the collection of diffraction images in high data-rate macromolecular crystallography this is an important trade-off. When designing the compressions used for the commercial release of the Dectris Pilatus detector in 2007 and the Dectris Eiger detector in 2013, the compressions chosen, byte-offset compression for the Pilatus and LZ4 for the Eiger, those choices were not of the highest compression ratios, but of moderate compression ratios achievable with the then-available cpus at the necessary frame rates. (Bernstein et al., 2016) (Donath et al., 2013). As image frame rates and cpu speeds increase, those tradeoffs need to be reconsidered. As of this writing the most efficient lossless compression is Zstandard.

S9. Compression level

In simplest terms, Zstandard higher compression levels use more memory and time to allow it to examine and optimize larger blocks of data. The highest compression levels use the highest memory to cache data to be scanned and compressed to a higher potential level. Since our data has an abundance of local repetition of image characteristics, peaks surrounded by background, the highest compression levels don't necessarily perform better, as they might for different types of images with more global structure and less repeated patterns.

Table S1 Compression ratio for two SAXS/WAXS reference data sets.

	**Compression Ratio**	
**Compression**	**Reference sample**	**Standard sample**
lz4	1.89	1.66
bslz4	6.65	4.67
slz4	4.99	3.98
zstd_2	5.03	3.61
bszstd_2	7.33	5.07
szstd_2	7.50	5.04
zstd_3	4.96	3.53
bszstd_3	7.53	5.19
szstd_3	7.48	5.09
zstd_4	5.65	3.97
bszstd_4	7.51	5.17
szstd_4	7.67	5.16
zstd_5	5.73	4.00
bszstd_5	7.52	5.18
szstd_5	7.69	5.16
zstd_6	5.68	4.01
bszstd_6	7.37	5.12
szstd_6	7.72	5.20
mean	6	4
